# TRPA1 channel mediates methylglyoxal-induced mouse bladder dysfunction

**DOI:** 10.3389/fphys.2023.1308077

**Published:** 2023-12-08

**Authors:** Akila L. Oliveira, Matheus L. Medeiros, Erick de Toledo Gomes, Glaucia Coelho Mello, Soraia Katia Pereira Costa, Fabíola Z. Mónica, Edson Antunes

**Affiliations:** ^1^ Department of Pharmacology, University of Campinas (UNICAMP), São Paulo, Brazil; ^2^ Department of Pharmacology, Institute of Biomedical Sciences, University of São Paulo (USP), São Paulo, Brazil

**Keywords:** urothelium, lamina propria, cystometry, void spot assay, MG-H1, glyoxalase

## Abstract

**Introduction:** The transient receptor potential ankyrin 1 channel (TRPA1) is expressed in urothelial cells and bladder nerve endings. Hyperglycemia in diabetic individuals induces accumulation of the highly reactive dicarbonyl compound methylglyoxal (MGO), which modulates TRPA1 activity. Long-term oral intake of MGO causes mouse bladder dysfunction. We hypothesized that TRPA1 takes part in the machinery that leads to MGO-induced bladder dysfunction. Therefore, we evaluated TRPA1 expression in the bladder and the effects of 1 h-intravesical infusion of the selective TRPA1 blocker HC-030031 (1 nmol/min) on MGO-induced cystometric alterations.

**Methods:** Five-week-old female C57BL/6 mice received 0.5% MGO in their drinking water for 12 weeks, whereas control mice received tap water alone.

**Results:** Compared to the control group, the protein levels and immunostaining for the MGO-derived hydroimidazolone isomer MG-H1 was increased in bladders of the MGO group, as observed in urothelium and detrusor smooth muscle. TRPA1 protein expression was significantly higher in bladder tissues of MGO compared to control group with TRPA1 immunostaining both lamina propria and urothelium, but not the detrusor smooth muscle. Void spot assays in conscious mice revealed an overactive bladder phenotype in MGO-treated mice characterized by increased number of voids and reduced volume per void. Filling cystometry in anaesthetized animals revealed an increased voiding frequency, reduced bladder capacity, and reduced voided volume in MGO compared to vehicle group, which were all reversed by HC-030031 infusion.

**Conclusion:** TRPA1 activation is implicated in MGO-induced mouse overactive bladder. TRPA1 blockers may be useful to treat diabetic bladder dysfunction in individuals with high MGO levels.

## Introduction

Diabetes Mellitus (DM) is a metabolic disease associated with high blood glucose levels and affects an increasing number of individuals worldwide (American Diabetes Association ADA, 2018). Life-threatening multi-organ complications associated with DM include cardiovascular diseases such as hypertension, stroke, and myocardial infarction, as well as conditions like retinopathy, peripheral neuropathy, and nephropathy (Wittig et al., 2019). Besides, diabetic bladder dysfunction (DBD) or diabetic cystopathy is a prevalent urological complication that refers to a group of bladder symptoms mainly found in long-standing and poorly controlled diabetic patients (Golbidi and Laher, 2010). Clinical symptoms of DBD can range from bladder overactivity, which includes urinary urgency, urge urinary incontinence, frequency, and nocturia during the early stages of the disease, to impaired bladder contractility during the late stages (Daneshgari et al., 2017; Song et al., 2022).

Elevated glycemic levels in diabetic patients lead to the accumulation of highly reactive dicarbonyl compounds in both plasma and urine, such as methylglyoxal (MGO) (Harkin et al., 2023). MGO is formed endogenously from 3-carbon glycolytic intermediates of glycolysis, despite it can be also generated as a byproduct of protein, lipid, and ketones (Kalapos, 1999; Thornalley et al., 1999; Lai et al., 2022). Once generated, MGO initiates post-translational modification of peptides and proteins, ultimately resulting in the generation of advanced glycation end products (AGEs), such as the arginine-derived hydroimidazolone (MG-H1). These AGEs interact with the cell membrane-anchored ligand receptor RAGE, triggering multiple signaling pathways that lead to production of inflammatory and pro-oxidant mediators (Schalkwijk and Stehouwer, 2020; Zhang et al., 2023). The enzymatic detoxification systems glyoxalase 1 (Glo1) and glyoxalase 2 (Glo2) play a pivotal role in converting MGO into its end-product, D-lactate (Rabbani and Thronalley, 2019). Recent studies revealed that supplementing the drinking water of both non-diabetic male and non-diabetic female mice with MGO for 4–12 weeks results in an overactive bladder phenotype, as assessed by voiding behavior and cystometric assays in awake and anesthetized animals, as well as by *in vitro* bladder contractility to electrical-field stimulation (EFS) and muscarinic receptor activation with carbachol (de Oliveira et al., 2020; Oliveira et al., 2021; Oliveira et al., 2022). Furthermore, diabetic obese ob/ob mice displaying high levels of MG-H1 and RAGE in bladder tissues also exhibit voiding dysfunction, suggesting that activation of the MGO-AGEs-RAGE pathway in the bladder wall contributes to the pathogenesis of diabetes-associated bladder dysfunction (Oliveira et al., 2023).

TRPA1 is embedded in the cell membrane and presents itself as a tetrameric form of a Ca^2+^ influx channel (Brauchi and Rothberg, 2020). Consequently, upon TRPA1 activation, the influx of Ca^2+^, along with other extracellular cations such as Na^+^ and H^+^, plays a pivotal role in triggering noxious responses, mostly associated with pain, cold, and itch (Gao et al., 2020). A large array of endogenously released chemical mediators, including nitric oxide, hydrogen sulfide, hydrogen peroxide, prostaglandin J, among others, as well as exogenous stimuli like cinnamaldehyde, allicin, allyl isothiocyanate, ligustilide, and acrolein can modulate the activity of TRPA1 channels (Gao et al., 2020). Additionally, MGO has been shown to activate the TRPA1 channel, particularly in diabetic neuropathic pain conditions (Ohkawara et al., 2012; Andersson et al., 2013; Huang et al., 2016; Griggs et al., 2017; Becker et al., 2023). The TRPA1 channel is expressed in the lower urinary tract, including nerve endings of the bladder wall (Andrade et al., 2011; Steiner et al., 2018; Andersson, 2019; de Oliveira et al., 2020; Kudsi et al., 2022; Zhao et al., 2022; Hayashi et al., 2023), and is believed to mediate bladder sensory transduction and contractility in diabetes (Philyppov et al., 2016; Blaha et al., 2019; Vanneste et al., 2021). TRPA1 mRNA expression has been detected in the bladder mucosa and bladder muscular layer, with upregulation seen in tissues obtained from patients with bladder outlet obstruction (Du et al., 2008). Given the implication of the TRPA1 channel in diabetes-related complications, we hypothesized that the TRPA1 channel plays an important role in the pathophysiology of urinary bladder dysfunction induced by chronic MGO intake. Therefore, the main objectives of this study were to identify alterations in TRPA1 expression in the bladder wall (mucosa and detrusor smooth muscle), and to evaluate the effects of the TRPA1 antagonist HC-030031 (Eid et al., 2008) on the *in vivo* and *in vitro* bladder dysfunction resulting from a 12-week treatment with MGO in female mice.

## Materials and methods

### Animals

Five-week-old female C57BL/6 mice weighing 19 ± 0.30 g at the beginning of the study were provided by Multidisciplinary Center for Biological Research on Laboratory Animal Science (CEMIB) of the State University of Campinas (UNICAMP, Sao Paulo, Brazil). Mice were housed in cages made of polypropylene with dimensions 30 × 20 × 13 cm placed in ventilated cage shelters with a constant humidity of 55% ± 5% and temperature of 24°C ± 1°C under a 12 h light-dark cycle. The animals (three mice per cage) were acclimated for 10 days before starting the treatments. Animals received standard food and filtered water *ad libitum*. Animal procedures and experimental protocols were approved by Ethics Committee in Animal Use of UNICAMP (CEUA-UNICAMP; protocol numbers 5443-1/2019 and 5842-1/2021). Animal studies follow the ARRIVE guidelines.

### Experimental design

We initially employed a randomization calculator, which is available at https://www.graphpad.com to allocate the mice into two groups, namely, Control group (*n* = 51) and MGO group (*n* = 51). In the MGO group, the animals received 0.5% MGO (Sigma Aldrich, Missouri, United States) in their drinking water for a duration of 12 weeks, as outlined in our previous study (Medeiros et al. 2021). The control group received only tap water. In the first part of this study, animals in the MGO group exhibiting voiding dysfunction through the void spot assay in filter paper were anesthetized using isoflurane and subsequently euthanized by cervical dislocation. Their bladders were then exposed and removed for the subsequent immunohistochemical and Western blotting assays, as described below. The same procedure was carried out in the control group. In the second phase of this study, filling cytometry in anesthetized animals and *in vitro* bladder contractility were selected to test the TRPA1 blocker HC-030031 in both control and MGO groups. The HC-030031 dose was set at 1 nmol/min for the 1-h intravesical infusion during cystometry or 10 µM for the *in vitro* assays.

### Void spot assay in filter paper

The objective of this test was to analyze the voiding behavior of animals that had been chronically administered MGO for 12 weeks. The analyzed parameters included the total void volume (the overall volume voided in 3 h), volume per void (average volume per void), and the total number of voids. Additionally, the number of voids was categorized based on volumes lower than 25 μL, volumes between 25 and 100 µL, and volumes higher than 100 µL. We also registered the distribution of voids in the center and corner of the filter paper to observe changes in spot distribution and normal micturition behavior, which involves animals seeking the corners of the cage, a phenomenon known as thigmotaxis (Hill et al., 2018). As such, the animals were individually housed in clean cages, each covered with a filter paper measuring 25 × 15 cm (qualitative filter paper 250 g Unifil^®^, Cod. 502.1250). Animals had no access to water but were provided with unrestricted access to food. The experiment was consistently conducted during a specific time window (9–10 a.m. to 12–13 p.m.), lasting for a duration of 3 h within the cages. The temperature of the room was maintained at 24 ± 1°C with a humidity level of 53% ± 1%. The animals were acclimated to the filter paper for 2 days, and void measurements were performed on the third day. At the conclusion of the assay, the animals were returned to their regular housing condition. Following the test, the voiding points were encircled with a pencil, and overlapping points were marked for subsequent quantification. The filter papers were dried and imaged using UV light (Photo-documenter Chemi-Doc, Bio-Rad, California, United States). The filter papers were then analyzed using the Fiji version of ImageJ Software (version 1.46r) (http://fiji.sc/wiki/index.php/Fiji), as previously described (Oliveira et al., 2021). Particles smaller than 0.20 cm^2^ (equivalent to 2 μL) were disregarded from consideration to minimize potential interference related to the paws or tail marks of the animals.

### Filling cystometry in anesthetized mice and TRPA1 antagonism with HC-030031

Filling cystometry was conducted following the method outlined as previously described (Oliveira et al., 2021). The animals were anesthetized using a rodent inhalation anesthesia system (Harvard Apparatus) and were maintained under anesthesia with a mixture of 2% isoflurane and 98% oxygen at a rate of 2 L/min. An abdominal incision was made to expose the urinary bladder. A PE10 catheter was carefully inserted into the apex of the bladder and fixed in place using a 6-0 nylon suture. Subsequently, the bladder was repositioned, and the surrounding musculature and skin were sutured closed. Following the completion of this surgical procedure, isoflurane anesthesia was discontinued, and an intraperitoneal injection of urethane (1.2 g/kg) was administered. The cannula was then connected to a 3-way tap, with one port linked to an infusion pump via a PE-10 catheter. Before initiating cystometry, a 10-min stabilization period was observed, after which continuous intravesical saline infusion was maintained at a rate of 0.6 mL/h for 1 h. Subsequently, the animals underwent continuous intravesical infusion for 1 h with either saline (0.01 mL/min), vehicle (0.001% DMSO) or the selective TRPA1 channel blocker HC-030031 (1 nmol/min; Catalogue No. H4415, Sigma-Aldrich, United States). Data acquisition was carried out using PowerLab system, and subsequent analyzes were performed using LabChart^®^ Software (ADInstruments Inc., Sydney, AU, https://www.adinstruments.com/products/labchart). The following parameters were assessed during the first hour of data acquisition: voiding frequency (number of voids/minute), bladder capacity (functional bladder capacity, which represents the volume infused during the intermicturition interval), voided volume (volume released during a voiding event), compliance (the ratio between capacity and threshold pressure, expressed in µl/mmHg), basal pressure (the minimum pressure observed between two voiding events), threshold pressure (the intravesical pressure immediately before voiding events), and maximum pressure (the highest bladder pressure recorded during a void). All the parameters were evaluated across all micturition cycles during the first hour of data acquisition. At the conclusion of the experimental protocols, the animals were euthanatized and disposed of accordingly.

### Exploring the effects of HC-030031 on the bladder contractions induced by electrical-field stimulation (EFS) and carbachol

At the conclusion of the 12-week MGO treatment, the animals were anesthetized with isoflurane, administered at a concentration exceeding 5%. Subsequently, cervical dislocation was performed to confirm euthanasia. The bladder was then removed and carefully divided into two strips, each representing an intact portion of the bladder. Strips were mounted in 10-mL organ baths containing Krebs-Henseleit solution, composed of the following constituents: 117 mM NaCl, 4.7 mM KCl, 2.5 mM CaCl_2_, 1.2 mM MgSO_4_, 1.2 mMKH_2_PO_4_, 25 mM NaHCO_3_ and 11 mM Glucose, pH 7.4. The solution was continuously oxygenated with a mixture of 95% O_2_ and 5% CO_2_. The tissues were allowed to equilibrate for 45 min under resting tension and were subsequently adjusted to a force of 5 mN. Changes in isometric force were recorded using a PowerLab system (ADInstruments Inc., Sydney, AU). After the stabilization period, one strip was incubated with the vehicle (0.001% DMSO) while the other was exposed to the selective TRPA1 antagonist HC-030031 (10 µM) for a duration of 30 min. Following the incubation period, EFS was applied using platinum ring electrodes placed between two strips, and connected to a stimulator (Grass Technologies, RI, United States). EFS was conducted at 80 V, with a pulse width of 1 ms pulse width, and trains of stimuli lasting 10 s were administered at varying frequencies ranging from 1 to 32 Hz, with 2-min intervals between each stimulation. Subsequently, cumulative concentration-response curves were generated for the muscarinic receptor agonist carbachol ranging from 1 nM to 100 μM (Sigma Aldrich, MI, United States). Non-linear regression analysis to determine the potency (pEC_50_) of carbachol was carried out using GraphPad Prism (GraphPad Software, Inc., CA, United States) with the constraint that F = 0. The concentration-response data were fitted to a logarithmic dose-response function with a variable slope in the form: E = Emax/([1 + (10c/10x) n] + F), where E is the effect of above basal, E_max_ is the maximum response produced by agonists; c is the logarithm of the pEC_50_, the concentration of drug that produces a half maximal response; x is the logarithm of the concentration of the drug; the exponential term, n, is a curve-fitting parameter that defines the slope of the concentration–response line, and F is the response observed in the absence of added drug. The contractile responses to EFS or carbachol were expressed as mN/mg.

### Immunohistochemistry for MG-H1 in bladder tissues

Bladder immunoperoxidase reactions were processed based on a previous study (Oliveira et al., 2022). Briefly, whole bladders were removed, immersed in 10% formalin fixative solution for 48 h, and embedded in paraffin. Five-micron sections were mounted onto aminopropyltriethoxysilane-coated glass slides. Sections were deparaffinized, rehydrated, and washed with 0.05 M Tris buffer solution (TBS) at pH 7.4. Subsequently, for antigen retrieval, sections were treated with 0.01 M citrate buffer containing 0.05% Tween-20 (pH 6.0) for 40 min at 98°C. Endogenous peroxidase activity was inhibited with 0.3% hydrogen peroxide (H_2_O_2_) solution. For blocking the non-specific sites, a 5% bovine serum albumin (BSA) solution containing 0.1% Tween-20 for 60 min was used. Sections were incubated with mouse monoclonal anti-MG-H1 primary antibody (1:90; Cell Biolabs, INC., Catalogue No. STA-011, San Diego, United States) diluted in TBS containing 3% BSA overnight at 4°C. Subsequently, sections were washed, and incubated with biotinylated goat anti-mouse IgG, avidin and biotinylated HRP (1:20; Catalogue No. EXTRA2, Sigma Aldrich, St Louis, MO, United States) following the manufacturer’s instructions. For detection of the immunostained area with MG-H1, a 3.3′diaminobenzidine solution (DAB; Catalogue No. D4293, Sigma Aldrich) was employed. As a negative control, a section was used in parallel to primary antibody omission. All slides were counterstained with hematoxylin and mounted for observation by microscopy. Representative images were acquired using a light microscope (OPTIKA ITALY B-1000 Series, OPTIKA S.r.l., Ponteranica, BG, Italy) equipped with a digital camera under a 4 × and 10 × objective.

### Immunohistochemistry for TRPA1 in bladder tissues

For immunohistochemistry of TRPA1, we followed the manufacturer’s instructions. The sections were deparaffinized, rehydrated, and washed with 1× phosphate buffered saline containing 0.1% Tween-20 (PBS-T). For antigen retrieval, the slides were boiled in 0.01 M sodium citrate buffer (pH 6.0) for 10 min, then cooled on bench top for 30 min. The sections were washed in distilled H_2_O (dH_2_O) three times for 5 min each, followed by a washing section 1 × PBS-T for 5 min. Endogenous peroxidase activity was inhibited with a 0.3% H_2_O_2_ solution. Each section with blocking solution (5% BSA) was blocked in 1× PBS-T solution for 1 h at room temperature. The blocking solution was then removed, and the primary antibody was diluted in 1× PBS-T with 5% BSA and added to each section and incubated overnight at 4°C with TRPA1 antibody (1:60; Catalogue No. 40763, Novus Biologicals, LLC, United States). The antibody solution was removed by washing 1 × PBS-T; and biotinylated secondary antibody diluted in 1 × PBS-T with 5% BSA was incubated for 30 min at room temperature (1:20; Catalogue No. EXTRA2, Sigma Aldrich, St Louis, MO, United States). The secondary antibody was removed by washing 1 × PBS-T and 100 µL streptavidin HRP reagent (1:20) was incubated for 30 min at room temperature in each section. For detection of the immunostained area with TRPA1, a 3.3′diaminobenzidine solution (DAB; Catalogue No. D4293, Sigma Aldrich) was employed, and sections were immersed in dH_2_0. The sections were counterstained in hematoxylin and mounted for observation by microscopy. Representative images were acquired using a light microscope (OPTIKA ITALY B-1000 Series, OPTIKA S.r.l., Ponteranica, BG, Italy) equipped with a digital camera under a 40 × objective.

### Western blot analysis of MG-H1, Glo1 and TRPA1 in bladder tissues

Total protein extracts were obtained from homogenized bladders using RIPA buffer (Catalogue No. R0278, Sigma-Aldrich, Darmstadt, Germany) containing protease inhibition cocktail (10 μL/mL; Catalogue No. P8340, Sigma-Aldrich, Darmstadt, Germany). The samples were incubated for 1 h at 4°C and then centrifuged at 12.000 g for 15 min at 4°C. Protein concentrations in the supernatants were determined using the DC Protein Assay Kit I (Catalogue No. 5000111EDU, BioRad, Hercules, CA, United States). An equal amount of protein (30 µg) from each sample was treated with 4× Laemmli buffer containing 355 mM of 2-mercaptoethanol (Catalogue No. 161-0747, BioRad, Hercules, CA, United States). The samples were heated in boiling water bath for 5 min and resolved by sodium dodecyl sulfate-polyacrylamide gel electrophoresis (SDS-PAGE). The proteins were then electrotransferred to a nitrocellulose membrane at 20 V for 20 min using a semi-dry device (Bio-Rad, Hercules, CA, United States). To reduce nonspecific protein binding, the membrane was pre-incubated overnight at 4°C in blocking buffer (0.5% non-fat dried milk, 10 mM Tris, 100 mM NaCl, and 0.02% Tween 20). Primary antibodies for mouse monoclonal MG-H1 (1:1000; Cell Biolabs, INC., Cat. No STA-011, San Diego, United States), Glo1 (Cat. No. ab96032, Abcam), TRPA1 (cat. No. 40763, Novus Biologicals, LLC, United States) and monoclonal β-actin peroxidase (1:50000, Catalogue No. A3854, Sigma-Aldrich, Darmstadt, Germany) were diluted in basal solution containing 3% BSA. These primary antibodies were validated and tested according to previous studies (Lee et al., 2016; Jandova and Wondrak, 2021; Smith et al., 2022; Chandrakumar et al., 2023; Luostarinen et al., 2023). The antibody was incubated overnight at 4°C, while the β-actin antibody was incubated for 1 h at room temperature. Subsequently, the membranes were incubated with the secondary antibody HRP-linked anti-rabbit IgG (1:5000; Catalogue No. 7074S, Cell Signaling Technology, Massachusetts, United States) diluted in basal solution for 1 h. Immunoreactive bands were detected using the Clarity Western ECL Substrate (Catalogue No. 1705061, BioRad, Hercules, CA, United States), an enhanced BioRad chemiluminescence system. Densitometry analysis was performed using the Image Lab Software Version 6.1 (BioRad, Hercules, CA, United States). The results were represented as the ratio of protein expression relative to β-actin.

### Assessment of Glo 1 activity in bladder tissues

The bladders were isolated, homogenized in 350 µL of PBS (pH 7.0), and then centrifuged at 2000 × g for 30 min at 4°C. Following centrifugation, the supernatant was removed and placed on ice. Glyoxalase I activity was assessed in duplicate using the Glyoxalase activity assay kit (Catalogue No. MAK114, Sigma-Aldrich, United States), following the manufacturer’s instructions. To normalize the results, the total protein content of the samples was determined in triplicated using the DC™ Protein Assay Kit II (Catalogue No. 5000112, Bio-Rad Laboratories, Inc. California, United States).

### Statistical analysis

The GraphPad Prism Version 6 Software (GraphPad Software, Inc., San Diego, CA, United States) was used for all statistical analysis. The parametric distribution of the data was assessed using the Shapiro test. Statistical difference between two groups was determined using Student’s unpaired *t*-test. One-way ANOVA followed by Dunnett’s multiple comparison test was used when comparing more than two groups with control group and one-way ANOVA followed by Tukey or Bonferroni’s test were used when comparing all groups together. All results are presented as the means ± standard error of the mean (SEM). Results with *p*-values lower than 0.05 were considered significant.

## Results

### Alterations in void spot patterns on the filter paper assay

**FIGURE 1:**
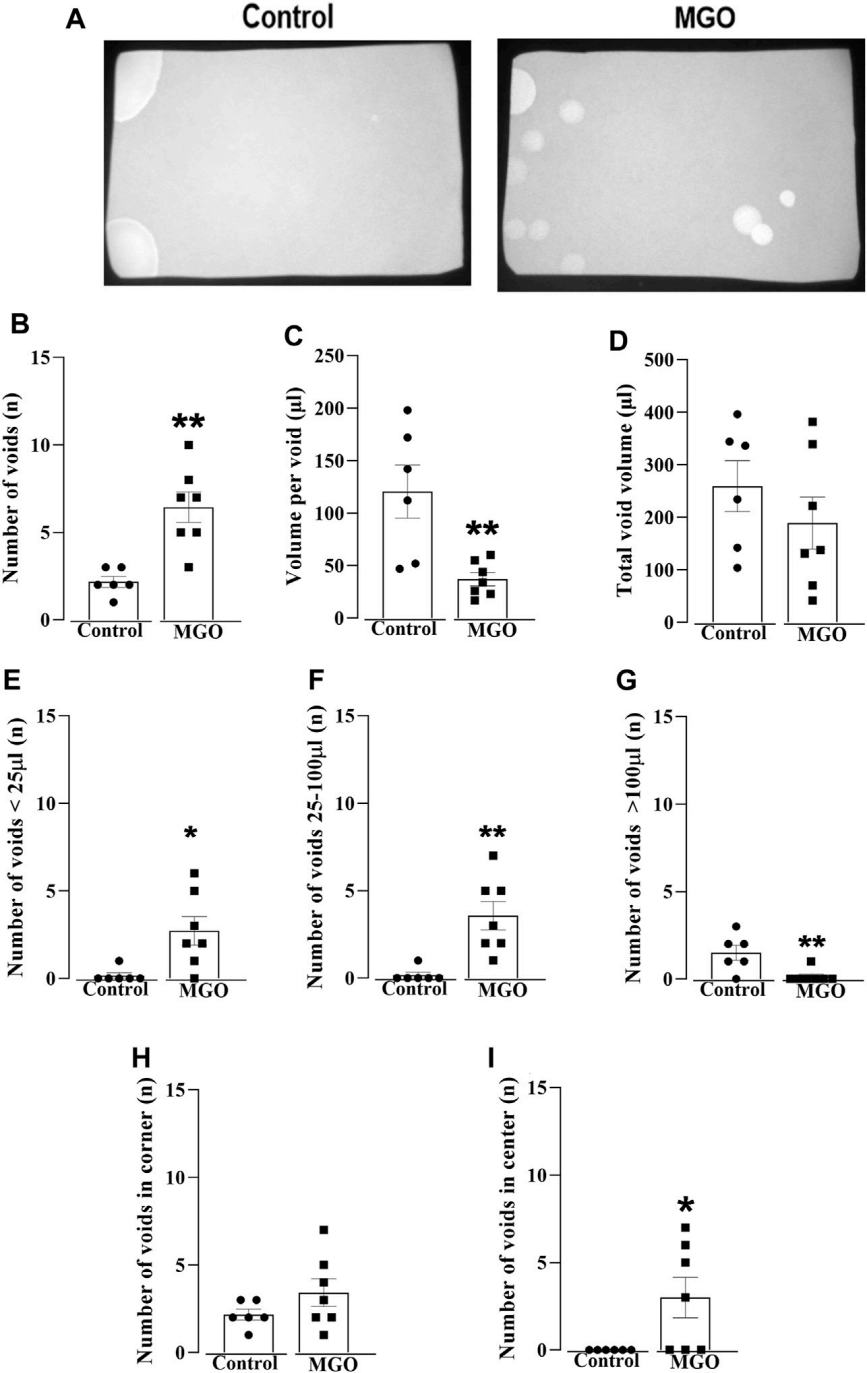
Void spot analysis in female mice exposed to 0.5% methylglyoxal (MGO) for 12 weeks. **(A)** displays representative images of the void spot assay in both the control group (receiving tap water alone) and the MGO-exposed groups. **(B–D)** show data on the number of voids, volume per void, and total void volume, respectively. The distribution of void spots across different volume ranges is shown in **(E)** (<25 µL), **(F)** (between 25 and 100 µL), and **(G)** (>100 µL). The number of voids in the corner and the center on the filter paper is illustrated in **(H,I)**, respectively. The data are expressed as the mean ± SEM (*n* = 6–7 animals per group). *p < 0.05, **p < 0.01 compared to control group (unpaired *t-*test).

The voiding dysfunction induced by a 12-week oral intake of MGO was initially screened in conscious mice using the void spot assay on filter paper (Figure 1). Figure 1A shows representative images of the void spot assays, revealing a significant increase in the number of spots in the MGO compared to the control group (*p* < 0.01; *n* = 6–7; Figure 1B). Furthermore, the volume per void (Figure 1C) was significantly reduced in the MGO group compared to the control group (*p* < 0.01), while no significant differences in total void volume were observed (Figure 1D). The number of voids based on their volume sizes revealed that mice treated with MGO exhibited a higher number of spots with volumes lower than 25 µL (*p* < 0.05; Figure 1E) and between 25 and 100 µL (*p* < 0.05; Figure 1F), along with a decreased number of spots with volumes greater than 100 µL (*p* < 0.01; Figure 1G) compared to control group. Notably, in the control group, void spots were essentially concentrated in the corner of the filter paper with no voids in the center, as expected under normal conditions. In contrast, the MGO-treated group exhibited a different distribution pattern of void spots, with voids now detected both in the center and the corner of the filter (Figures 1H, I).

### Protein levels and immunohistochemistry for MG-H1 in the bladder

**FIGURE 2:**
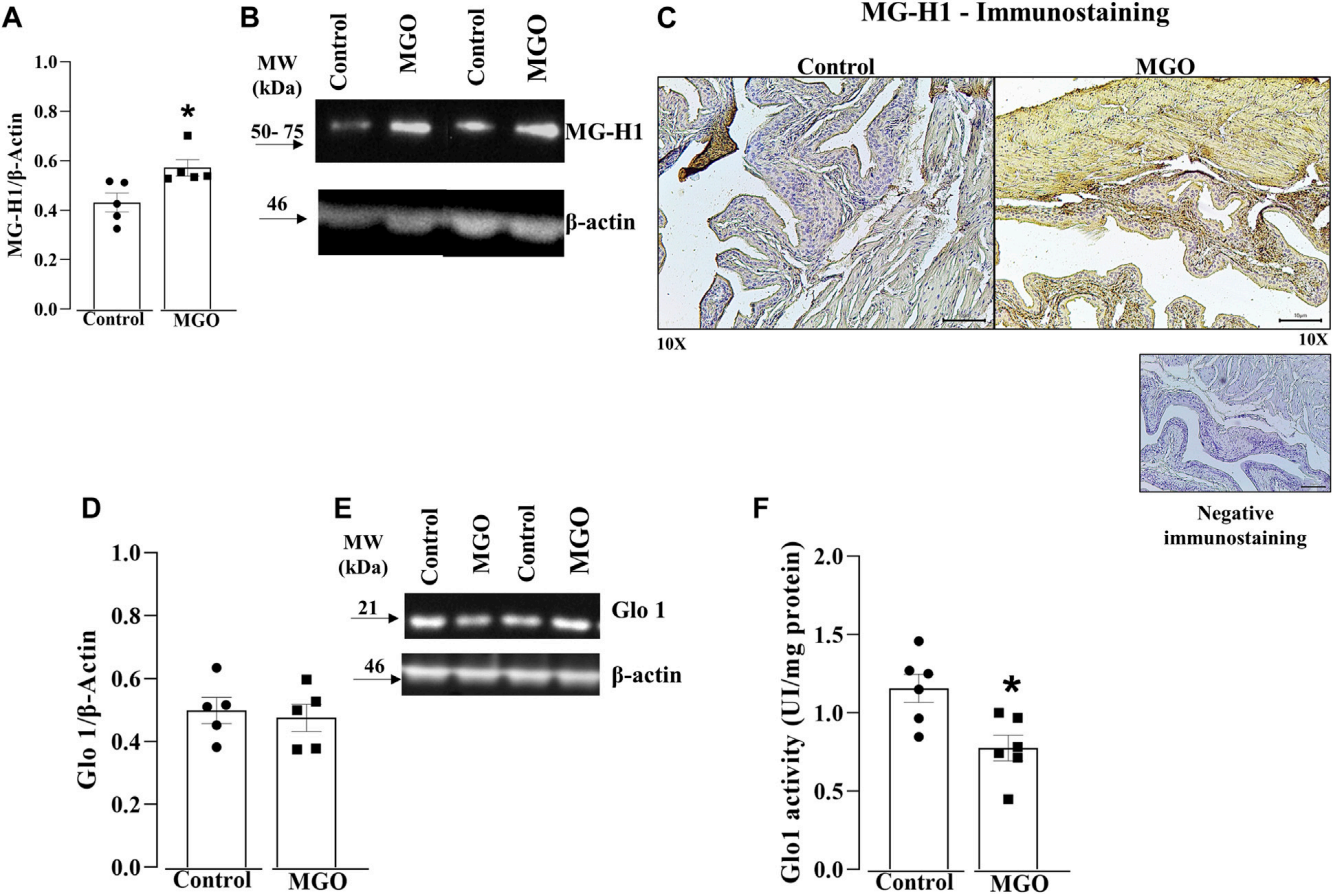
Quantification of methylglyoxal (MGO)-derived hydroimidazolone MG-H1 and glyoxalase 1 (Glo1) in the bladders of MGO-treated mice. **(A,B)** display densitometry analyses and representative Western blots of MG-H1, respectively. **(C)** depicts immunohistochemistry for MG-H1, demonstrating negative staining (absence of primary antibody binding) and positive immunostainings in control and MGO groups. In the control group, there is a weak positive staining observed in the urothelium, while in the MGO group, a strong positive staining is observed in both the urothelium and detrusor smooth muscle. In **(C)**, black bars represent a scale of 10 μm (×10 objectives). **(D,E)** display the protein expression, whereas **(F)** shows the Glo1 activity. The data are expressed as mean ± SEM (*n* = 5–6 animals per group). *p < 0.05 compared to control group (unpaired *t-*test).

Higher protein levels of MG-H1 were found in MGO compared to control group (*p* < 0.05; Figures 2A, B; Supplementary Figure S1A). Immunohistochemical assays showed a marked MG-H1 immunostaining in both the urothelium and detrusor smooth muscle layers of MGO-treated mice, whereas only minimal MG-H1 immunostaining intensity was detected in the urothelium of the control group (Figure 2C).

### Protein levels and enzyme activity of Glo1 in the bladder

Protein levels of Glo1 in bladder tissues did not significantly differ between the control and MGO groups (Figures 2D, E; Supplementary Figure S1B). However, MGO treatment led to a significant decrease in Glo1 activity in bladder tissues compared to control group (Figure 2F).

### Levels of TRPA1 are enhanced in the bladder of MGO-Treated mice

**FIGURE 3:**
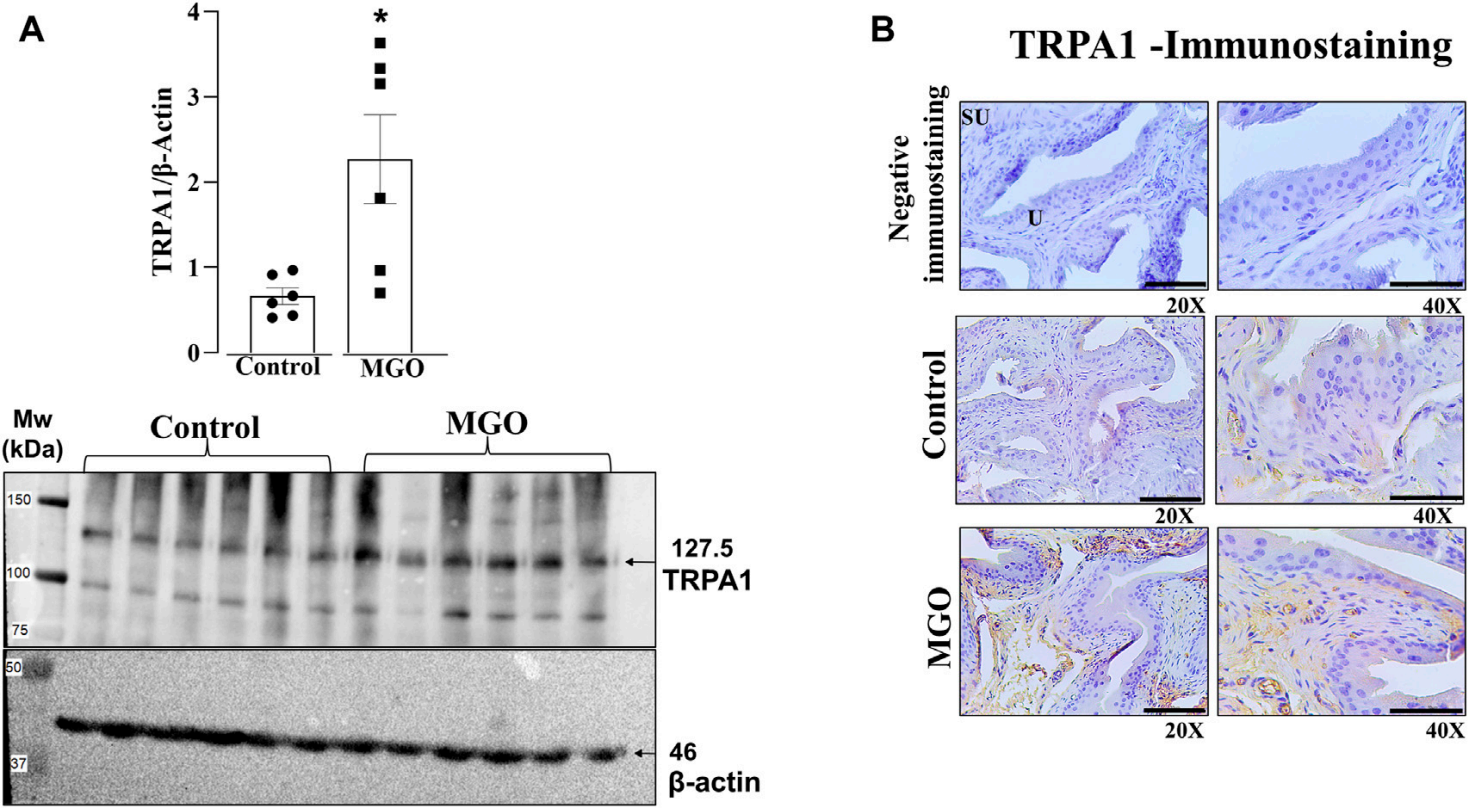
Protein expression of TRPA1 in the bladders of mice was assessed following a 12-week treatment with 0.5% methylglyoxal (MGO) or in control animals receiving tap water alone. **(A)** illustrates the results of protein expression analysis using Western Blotting analysis. **(B)** displays the immunohistochemistry for TRPA1 in bladder, showing negative immunostaining (absence of the antibody signal), and positive immunostainings in control and MGO groups. Positive immunostaining is observed in lamina propria and urothelial cells. The black bars in **(B)** represent a scale of 10 μm, as viewed under ×20 and ×40 objectives. In **(A)**, data are expressed as mean ± SEM (*n* = 7–8 animals per group). *p < 0.05 compared to control group (unpaired *t-*test).

Western blotting and immunohistochemistry assays were carried out in bladder tissues obtained from control and MGO-treated mice to investigate the expression of TRPA1. The results revealed a marked increase in TRPA1 protein levels in the bladder of MGO-treated mice compared to control groups (*p* < 0.05; Figure 3A; Supplementary Figure S2). In both control and MGO-treated groups, TRPA1 immunostaining was detected in the bladder mucosa, including the lamina propria and urothelial cells. Notably, the MGO-treated group exhibited a substantially higher intensity of TRPA1 immunostaining (Figure 3B). It is worthing mentioning that no TRPA1 immunostaining was detected in the detrusor smooth muscle layer of either group.

### Intravesical infusion of HC-030031 reverses cystometric alterations in MGO-Treated mice

**FIGURE 4:**
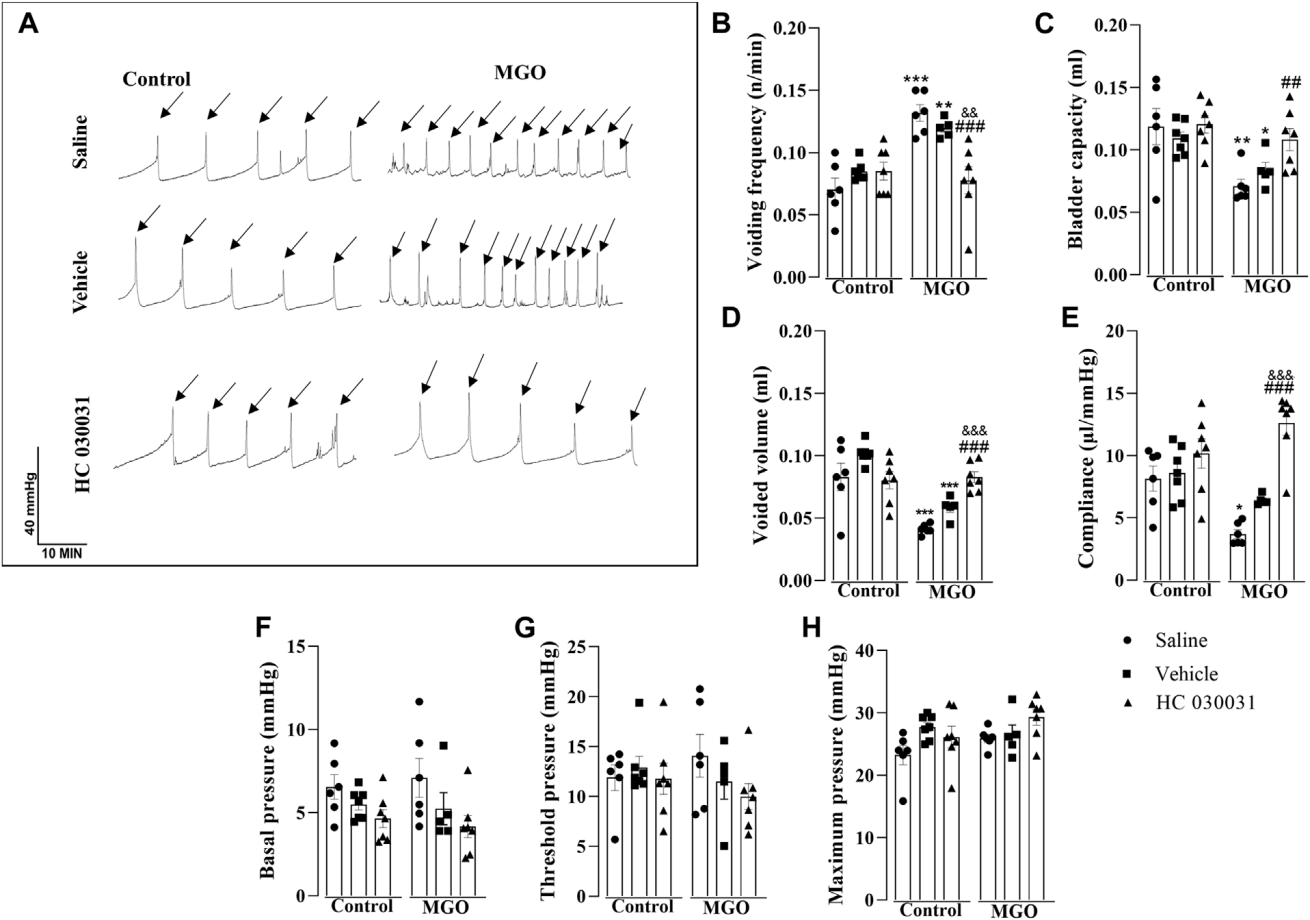
Effect of continuous infusion of the selective TRPA1 antagonist HC-030031 on cystometric changes in mice following 12-week treatment with 0.5% methylglyoxal (MGO) or control animals receiving tap water alone. In control and MGO groups, animals were infused continuously with saline (0.01 mL/min), vehicle (0.001% DMSO) or HC-030031 (1 nmol/min) for 1 h. **(A)** shows representative cystometric tracings from each sub-group, with arrows indicating micturition peaks. **(B–H)** show data on voiding frequency, bladder capacity, voided volume, compliance, basal pressure, threshold pressure and maximum pressure, respectively. All data are expressed as mean ± SEM (*n* = 5–7 animals per group). *p < 0.05, **p < 0.01, ***p < 0.001 compared to respective control group; ^##^ *p* < 0.01, ^###^ *p* < 0.001 compared to saline infusion in MGO group; ^&&^ *p* < 0.01, ^&&&^ *p* < 0.001 compared vehicle infusion in MGO group (one-way ANOVA followed by Dunnett’s multiple comparisons test for comparison to the control group and Bonferroni’s multiple comparisons test to compare all groups).

In order to evaluate the role of TRPA1 on voiding dysfunction induced by chronic MGO intake, we moved to the model of filling cystometry assays in anesthetized mice, which allowed us testing the TRPA1 antagonist HC-030031 by intravesical infusion on the resulting voiding dysfunction. Control and MGO-treated mice were intravesically infused with HC-030031 (1 nmol/min), saline or vehicle (0.001% DMSO; *n* = 5–7 animals per group). Compared to control group, a different pattern of voiding was found in MGO groups infused with either saline or vehicle, as characterized by a significantly higher voiding frequency (Figures 4A, B), paralleling significant reductions of bladder capacity (Figure 4C), voided volume (Figure 4D) and compliance (Figure 4E). The basal pressure (Figure 4F), threshold pressure (Figure 4G), and maximum pressure (Figure 4H) did not significantly differ between control and MGO groups. In MGO-treated mice, the infusion of HC-030031 almost completely reversed the changes in voiding frequency, bladder capacity, voided volume, and compliance. The basal pressure, threshold pressure, and maximum pressure remained unaltered. Infusion of HC-030031 at the same dose into the control group did not have a significant effect in any of the cystometric parameters. There were no statistical differences in any parameter between the saline and vehicle infusions in both the control and MGO groups.

### HC-030031 reduces the *in vitro* bladder contractility of MGO-Treated mice

**FIGURE 5:**
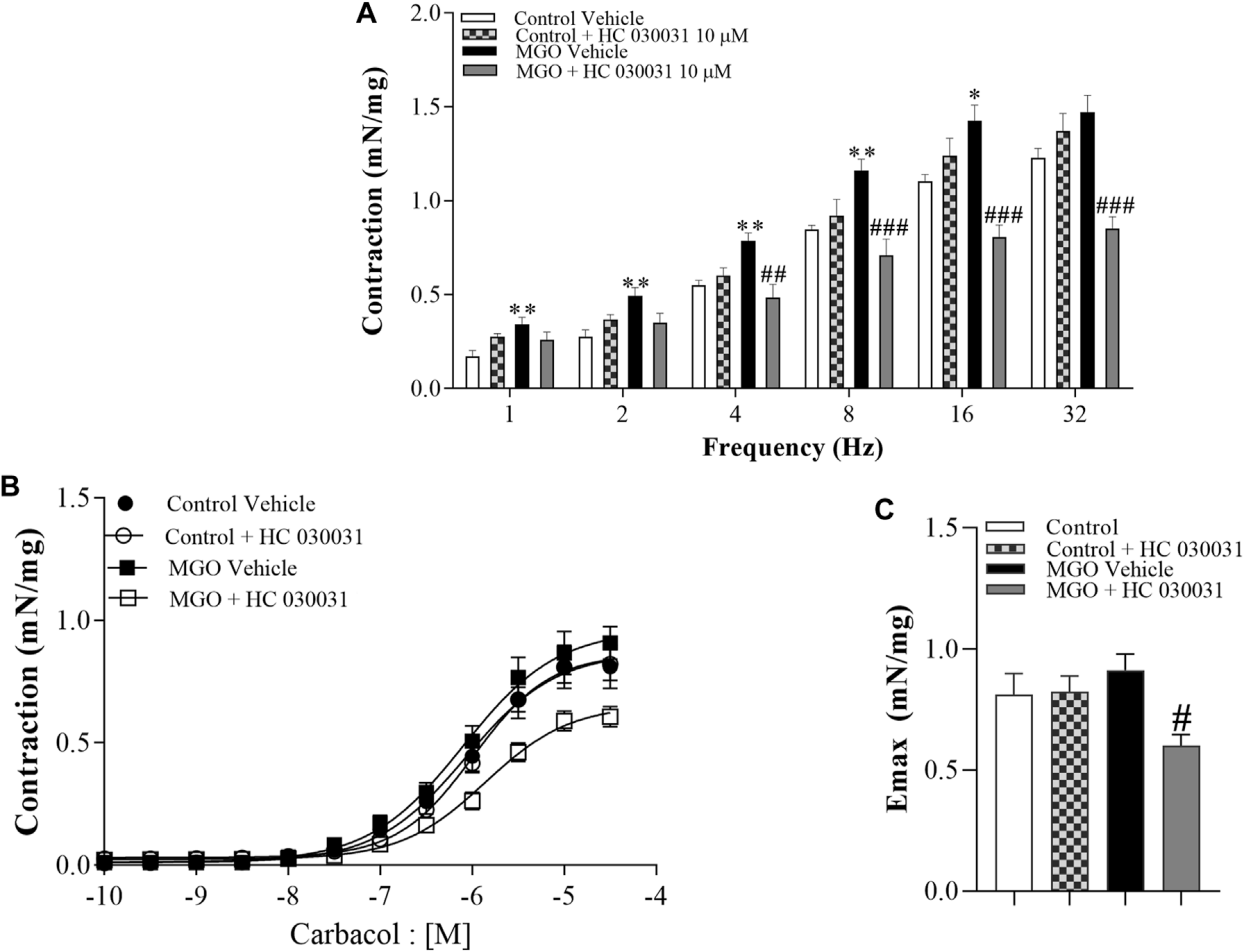
Effects of the selective TRPA1 antagonist HC-030031 on the contractile responses induced by electrical-field stimulation (EFS) and the muscarinic agonist carbachol in intact bladder strips obtained from mice treated with 0.5% methylglyoxal (MGO, 12 weeks) or tap water (control group). **(A)** illustrates the contractions induced by EFS at frequencies ranging from 1 to 32 Hz in both the control and MGO-treated groups in the presence of vehicle (0.001% DMSO) or HC-030031 (10 μM, 30 min). **(B)** displays the contractions induced by carbachol at concentrations ranging from 10^−10^ to 3 × 10^−5^ M in both the control and MGO-treated groups, in the presence of vehicle or HC-030031. **(C)** shows the maximal responses (E_max_) to carbachol in all experimental groups. The data are expressed as mean ± SEM (*n* = 7 animals per group). *p < 0.05, **p < 0.01 compared to corresponding control vehicle group. ^#^ *p* < 0.05 ^##^ *p* < 0.01 ^###^ *p* < 0.001 compared to respective MGO vehicle group (One-way ANOVA followed by the Tukey).

The contractile responses elicited by EFS and carbachol were examined in intact bladder strips (Figure 5). Electrical-field stimulation at a frequency of 1–32 Hz produced frequency-dependent bladder contractions in both the control and MGO groups. However, the contractions in the MGO were significantly higher than those in the control group, as evidenced at frequencies of 1–16 Hz (Figure 5A). In the control group, the prior incubation of bladder strips with HC-030031 (10 μM, 30 min) had no significant effect on EFS-induced contractions. Conversely, in the MGO group, HC-030031 completely restored the contractile responses to the levels observed in the control group (Figure 5A).

Addition of carbachol (10^−10^ to 3 × 10^−5^ M) produced concentration-dependent bladder contractions with no differences between MGO and control groups (Figure 5B). However, in the MGO group, HC-030031 (10 μM, 30 min) significantly reduced the carbachol-induced contractions, whereas in the control group HC-030031 had no significant effect (Figures 5B, C). The pEC_50_ values for carbachol did not significantly differ between groups, with values of 6.06 ± 0.11 for control + vehicle, 5.98 ± 0.06 for control + HC-030031, 6.09 ± 0.10 for MGO + vehicle, and 5.85 ± 0.08 for MGO + HC-030031.

## Discussion

Chronic exposure to MGO induces an overactive bladder phenotype in mice, as observed in previous studies (de Oliveira et al., 2020; Oliveira et al., 2021; Oliveira et al., 2022). TRPA1 is expressed in human (Du et al., 2008), rat (Streng et al., 2008; Andrade et al., 2011) and mouse bladders (de Oliveira et al., 2020) and is upregulated in pathological conditions such as bladder outlet obstruction and spinal cord injury. We tested here the hypothesis that TRPA1 activation in bladder tissues contributes, at least in part, to MGO-induced bladder dysfunction in female mice.

Initially, we assessed voiding dysfunction in MGO-treated mice using the void spot on the filter paper assay (Hill et al., 2018). The data confirmed the presence of an overactive phenotype in MGO-treated male mice (de Oliveira et al., 2020), as evidenced by an increased number of urine spots together with a reduction in voided volume per void. Furthermore, the MGO-treated group exhibited a higher number of voids with small volumes (less than 25 µL and between 25 and 100 µL), primarily concentrated in the center of the filter paper. Despite the bladder weight increases by about of 20% in female mice treated with MGO, the water consumption does not significantly differ between MGO and vehicle groups (Oliveira et al., 2022), suggesting that alterations of voiding behavior in MGO-treated mice does not reflect mechanisms dependent on fluid intake, as observed in streptozotocin-injected animals, leptin-deficient ob/ob mice, and leptin receptor-deficient db/db mice (Suriano et al., 2021; Yesilyurt et al., 2022). Subsequently, we evaluated the protein levels and immunostaining of the MGO adduct MG-H1 in bladder tissues of both controls and MGO-treated mice. Compared to control group, the MGO-treated mice displayed higher protein levels and increased immunostaining of the MG-H1 in both the urothelium and detrusor smooth muscle. Dicarbonyl stress is characterized by an abnormal glycolytic overload and elevated cellular MGO concentration, which is critically regulated by Glo1 activity, one of the primary enzymes involved in MGO detoxification (Rabbani and Thornalley, 2019; He et al., 2020). In the present study, the protein expression of Glo1 in bladder tissues remained unchanged following MGO treatment. However, Glo1 activity was significantly reduced in the MGO compared to control group. This reduction in Glo1 activity in MGO-treated mice is likely attributed to the accumulation of MGO in the bladders, as evidenced by the higher levels of the MGO adduct MG-H1, consistent with the presence of a true dicarbonyl stress in bladder tissues of these animals.

We then explored the role of TRPA1 in MGO-induced voiding dysfunction. Higher levels of TRPA1 protein were found in the bladder tissues of MGO-treated mice as compared to control group. Additionally, intense TRPA1 immunostaining was detected in the lamina propria of the MGO group, despite urothelial cells expressing TRPA1 being also observed. Nevertheless, no TRPA1 immunostaining was detected in the detrusor smooth muscle layer. In a separate set of experiments, cystometric assays were conducted in anaesthetized control and MGO-treated mice, with and without intravesical infusion of the TRPA1 blocker HC-030031, vehicle (0.001% DMSO) or saline. Methylglyoxal-treated mice displayed increased voiding frequency along with reductions of voided volume, bladder capacity, and compliance, consistent with the voiding alterations observed in conscious animals using the void spot assay. Importantly, these MGO-induced cystometric alterations were all reversed by intravesical infusion of HC-030031, indicating that TRPA1 activation in the urothelium plays a role in the machinery leading to overactive bladder.

Next, we assessed *in vitro* bladder contractions in response to EFS and carbachol in both control and MGO-treated mice. EFS-induced bladder contractions are chiefly mediated by acetylcholine release from parasympathetic fiber terminals, acting through the activation of post-synaptic muscarinic M_3_ receptor in detrusor smooth muscle (Fry et al., 2010; Sellers and Chess-Williams, 2012). Muscarinic M3 receptors coupled to phospholipase C-dependent signals mediate bladder contractions via generation of the second messenger inositol triphosphate (IP_3_), which activates the IP receptor to release Ca^2+^ from internal stores, in addition to extracellular Ca^2+^ influx secondary to L-type Ca^2+^ channel opening (Abrams et al., 2006; Frazier et al., 2008; Leiria et al., 2011). Nerve-mediated ATP release is also observed in mouse detrusor smooth muscle, which is said to mediate the atropine-resistant bladder contraction through P2X1 receptor activation (Tsai et al., 2012; Hao et al., 2019; McCarthy et al., 2019; Chakrabarty et al., 2022). A crosstalk between the purinergic and cholinergic transmitter systems, where ATP appears to induce the release of acetylcholine has also been reported (Stenqvist et al., 2020). We then assessed *in vitro* bladder contractions in response to EFS and carbachol in both control and MGO-treated mice. A previous study of our group showed that the contractile responses to the selective muscarinic agonist carbachol in MGO-treated mice remain unchanged intact bladder strips, but mucosal removal significantly increases in carbachol-induced bladder contractions (Oliveira et al., 2022). Interestingly, however, in the present study using intact bladder strip preparations, HC-030031 significantly reduced the carbachol-induced contractions in MGO-treated mice. On the other hand, MGO exposure was described to significantly enhance the contractile responses to both EFS and α,β-methylene ATP (a P2X1 purinergic receptor agonist) independently of the presence or not of urothelium (Oliveira et al., 2022). In the present study, the higher contractile response to EFS in bladders of MGO-treated mice was normalized by prior incubation with HC-030031. These data are indicative that MGO exposure via TRPA1 activation leads to enhancement of purinergic over cholinergic neurotransmission in the bladder. Of interest, interaction of TRPA1 and purinergic P2X receptors has been proposed to explain the pain pathophysiology in models of formalin-induced behavioral nociceptive responses in the rat (Krimon et al., 2013) and intracolonic administration of a low dose mustard oil in mice (Gonzalez-Cano et al., 2021). However, future experiments exploring the P2X1 purinergic component of the EFS-induced excitatory transmission might help to shed some light on the potential interactions of P2X and TRPA1 receptors in bladder of MGO-treated mice.

Collectively, our data from molecular and functional (*in vivo* and *in vitro*) studies support an important role of urothelial TRPA1 in modulating the bladder contractile responses after exposure to MGO. A previous study carried out in diabetic rats showed an increased mRNA expression of TRPA1 in dorsal root ganglia (DRG) that innervate the bladder and TRPA1 activation enhances the amplitude of EFS-induced detrusor smooth muscle contractions through mechanisms related to the activation of the tachykininergic and prostanoid systems (Philyppov et al., 2016). Increased TRPA1 expression was also seen in the bladders of insulin-resistant obese Zucker rats, but EFS-induced bladder contractions were instead reduced being this reduction attributed to excessive oxidative stress and downregulation of the cysthathionine-*γ*-lyase (CSE)/hydrogen sulfide (H_2_S) pathway (Blaha et al., 2019). TRPA1 has been proposed to serve as an oxidative stress sensor (Yamamoto and Shimizu, 2016; Anraku, 2022), and exposure to MGO increases the production of reactive-oxygen species (ROS) that in turn leads to activation of Rho kinase system in detrusor smooth muscle, ultimately promoting detrusor overactivity (Oliveira et al., 2022). Therefore, further investigation is needed to identify the intracellular signal mediated by MGO that upregulates TRPA1 in bladder urothelium and lamina propria, thereby enhancing detrusor smooth muscle contractility.

## Conclusion

In conclusion, this study shows that prolonged exposure to MGO in mice results in elevated levels of the advanced glycation end product MG-H1 in bladder tissues, inducing an upregulation of TRPA1 expression in the mucosal layer (lamina propria and urothelium). The effective blockade of TRPA1 with HC-030031 efficiently mitigated MGO-induced overactive bladder and detrusor hyperactivity. TRPA1 antagonists could potentially serve as a valuable therapeutic approach for managing diabetic bladder dysfunction in individuals with high MGO levels.

## Data Availability

The raw data supporting the conclusion of this article will be made available by the authors, without undue reservation.
