# Supplementary material for: TRPA1 channel mediates methylglyoxal-induced mouse bladder dysfunction
Source: Front Physiol. 2023 Dec 8;14:1308077. doi: 10.3389/fphys.2023.1308077 (PMC10739337; doi:10.3389/fphys.2023.1308077)
Supplement: Supplementary file 1 [file DataSheet1.PDF]

Supplementary Material

TRPA1 Channel Mediates Methylglyoxal-Induced Bladder Dysfunction

Akila L. Oliveira, Matheus L. Medeiros, Erick de Toledo Gomes, Glaucia Coelho Mello, Soraia Katia Pereira Costa, Fabíola Z. Mónica and Edson Antunes,

*** Correspondence:** Corresponding Author: eantunes@unicamp.br (alternative e-mail: edson.antunes@uol.com.br)

1 Supplementary Figures

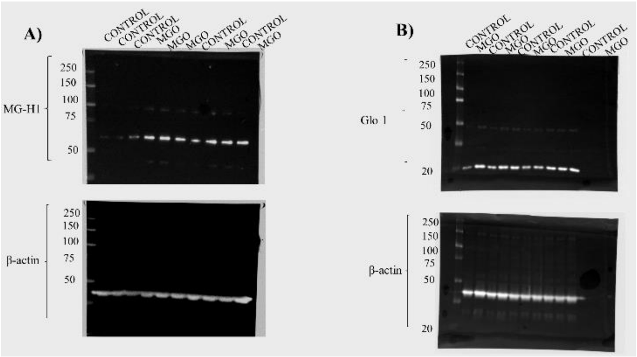

Supplementary Figure 1. Western blotting analyzes of methylglyoxal (MGO)-derived hydroimidazolone MG-H1 (A) and glyoxalase 1 (Glo1) in the bladders of MGO-treated mice (B) in comparison with control groups. Each antibody was used on one membrane, without repeating the membranes and normalized to β-actin.

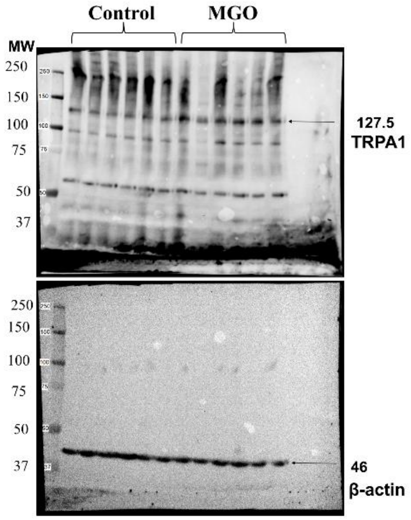

Supplementary Figure 2. Western blotting analyzes of TRPA1 in the bladders of methylglyoxal (MGO)-treated mice compared with control group. Each antibody was used on one membrane, without repeating the membranes, and normalized to β-actin.
